# Supplementary material for: Understanding Graph Isomorphism Network for rs-fMRI Functional Connectivity Analysis
Source: Front Neurosci. 2020 Jun 30;14:630. doi: 10.3389/fnins.2020.00630 (PMC7344313; doi:10.3389/fnins.2020.00630)
Supplement: Supplementary file 1 [file Data_Sheet_1.PDF]

Supplementary Material

1 EXPRESSIVE EXPLANATION OF THE GIN OPERATION

This section is devoted to explaining the GIN operation (9) in a more expressive manner. For that purpose, we consider a small graph with four nodes ($N = 4$) and four edges (Figure 3). An example one-hot input node feature matrix $\boldsymbol{X}$ and the adjacency matrix $\boldsymbol{A}$ are defined as following (Figure 3. A),

$$\boldsymbol{X}^{(0)} = \begin{bmatrix} 1 & 0 & 0 & 0 \\ 0 & 1 & 0 & 0 \\ 0 & 0 & 1 & 0 \\ 0 & 0 & 0 & 1 \end{bmatrix}$$

$$\boldsymbol{A} = \begin{bmatrix} 0 & 1 & 1 & 0 \\ 1 & 0 & 1 & 1 \\ 1 & 1 & 1 & 0 \\ 0 & 1 & 0 & 0 \end{bmatrix}.$$

Next, each node feature is multiplied by $1 + \epsilon^{(0)}$, and the neighboring node features are summed, as in the brackets of the (9) (Figure 3. B). In this example, we set the learnable parameter $\epsilon^{(0)} = 0.1$ to obtain the aggregated feature matrix

$$\boldsymbol{R}^{(0)} = \begin{bmatrix} 1.1 & 1 & 1 & 0 \\ 1 & 1.1 & 1 & 0 \\ 1 & 1 & 1.1 & 1 \\ 0 & 0 & 0 & 1.1 \end{bmatrix}.$$

The aggregated feature matrix $\boldsymbol{R}^{(0)}$ is mapped through the MLP and then the ReLU nonlinearity (Figure 3. C, D).

Here we set the example MLP weight matrix $\boldsymbol{W}^{(0)}$ as

$$\boldsymbol{W}^{(0)} = \begin{bmatrix} 0.1 & -0.2 & -0.3 & 0.4 \\ -0.1 & 0.2 & -0.3 & 0.4 \\ 0.4 & 0.3 & 0.2 & -0.1 \\ -0.4 & 0.3 & 0.2 & -0.1 \end{bmatrix}.$$

to obtain the next layer feature matrix $\boldsymbol{X}^{(1)}$ as

$$\boldsymbol{X}^{(1)} = \sigma\left(\begin{bmatrix} 0.11 & -0.3 & -0.2 & 0 \\ -0.1 & -0.33 & 0.2 & 0 \\ 0.4 & 0.2 & 0.33 & -0.1 \\ 0 & 0 & 0 & -0.11 \end{bmatrix}\right)$$
$$= \begin{bmatrix} 0.11 & 0 & 0 & 0 \\ 0 & 0 & 0.2 & 0 \\ 0.4 & 0.2 & 0.33 & 0 \\ 0 & 0 & 0 & 0 \end{bmatrix}.$$

Like the above example process, same operations are applied to the mapped node feature of the k-th layer $\boldsymbol{X}^{(k)}$ in each layers of the GIN (9).

2 LINEAR DEPENDENCE OF THE NODE FEATURES REDUCES THE DISCRIMINATIVE POWER OF THE GIN

We demonstrate the importance of embedding the node as a one-hot vector, based on the idea of (Xu et al., 2018a). In terms of graph classification tasks, (Xu et al., 2018a) has shown that the GIN can be as discriminative as the Weisfeiler-Lehman (WL) test. WL test is a test for solving the graph isomorphism problem, where graph isomorphism means whether two seperate graphs are topologically identical. Conditions for achieving this maximum discriminative power is also provided by (Xu et al., 2018a), which are that if the `AGGREGATE`, `COMBINE`, and `READOUT` mappings of the GNN are injective, then the GNN is as powerful as the WL test. One issue here is that `AGGREGATE` is usually implemented as sum operation, so if input node feature vectors are not linearly independent, it leads to a non-injective mapping.

For example, assume that two distinct node features are embedded into two vectors $\boldsymbol{p}_1^{(0)}$ and $\boldsymbol{p}_2^{(0)}$, respectively. If $\boldsymbol{p}_1^{(0)}$ and $\boldsymbol{p}_2^{(0)}$ are linearly dependent, then there exist positive integers a and b such that

$$\boldsymbol{p}_1^{(0)} = \frac{a}{b}\boldsymbol{p}_2^{(0)} \tag{S1}$$

Consider one simple graph G_1 that comprise $b+1$ vertices with all features as $\boldsymbol{p}_1^{(0)}$ where there exists edges between the first node and the others, that is

$$\boldsymbol{X}^{(0)} = \underbrace{\begin{bmatrix} \boldsymbol{p}_1^{(0)} & \boldsymbol{p}_1^{(0)} & \cdots & \boldsymbol{p}_1^{(0)} \end{bmatrix}}_{b+1}^{\top},$$

$$\boldsymbol{A} = \begin{bmatrix} 0 & 1 & \cdots & 1 & 1 \\ 1 & 0 & \cdots & 0 & 0 \\ \vdots & \vdots & \ddots & \vdots & \vdots \\ 1 & 0 & \cdots & 0 & 0 \\ 1 & 0 & \cdots & 0 & 0 \end{bmatrix}$$

Then the node feature vectors of the next layer from (13) is

$$\begin{aligned} \boldsymbol{r}_1^{(1)} &= c^{(1)}\boldsymbol{p}_1^{(0)} + b\boldsymbol{p}_1^{(0)} \\ \boldsymbol{r}_v^{(1)} &= c^{(1)}\boldsymbol{p}_1^{(0)} + \boldsymbol{p}_1^{(0)}, \quad v = 2, 3, \cdots, b+1 \end{aligned} \tag{S2}$$

Now, consider another simple graph G_2 that comprise $a+1$ vertices with a feature $\boldsymbol{p}_1^{(0)}$, and the other features as $\boldsymbol{p}_2^{(0)}$ where there exists edges between the first node and the others, that is

$$\boldsymbol{X}^{(0)} = \underbrace{\begin{bmatrix} \boldsymbol{p}_1^{(0)} & \boldsymbol{p}_2^{(0)} & \cdots & \boldsymbol{p}_2^{(0)} \end{bmatrix}}_{a+1}^{\top},$$

$$\boldsymbol{A} = \begin{bmatrix} 0 & 1 & \cdots & 1 & 1 \\ 1 & 0 & \cdots & 0 & 0 \\ \vdots & \vdots & \ddots & \vdots & \vdots \\ 1 & 0 & \cdots & 0 & 0 \\ 1 & 0 & \cdots & 0 & 0 \end{bmatrix}$$

Then the node feature vectors of the next layer from (13) is

$$\begin{aligned} \boldsymbol{r}_1^{(1)} &= c^{(1)}\boldsymbol{p}_1^{(0)} + a\boldsymbol{p}_2^{(0)} \\ \boldsymbol{r}_v^{(1)} &= c^{(1)}\boldsymbol{p}_2^{(0)} + \boldsymbol{p}_2^{(0)}, \quad v = 2, 3, \cdots, a+1 \end{aligned} \tag{S3}$$

We can now see that the first layer node embeddings of the G_1 (S2) and the G_2 (S3) are identical given (S1),

$$\begin{aligned} \boldsymbol{r}_1^{(1)} &= c^{(1)}\boldsymbol{p}_1^{(0)} + b\boldsymbol{p}_1^{(0)} \\ &= c^{(1)}\boldsymbol{p}_1^{(0)} + a\boldsymbol{p}_2^{(0)}. \end{aligned}$$

In this case, regardless of the MLP, the embedding of the first node of the G_1 and the G_2 are not discriminative with the GIN (9).

Thus, it can be said that it is more practical to make the set of input feature vectors linearly independent to each other. By embedding each separate ROIs into a one-hot vector encoding, it can be ensured that the input features are orthogonal, needless to say linearly independent, to each other. Moreover, one-hot vector encoding leads to a more interpretable Grad-CAM saliency map as in (24).
